# *Ox40-Cre–*mediated deletion of BRD4 reveals an unexpected phenotype of hair follicle stem cells in alopecia

**DOI:** 10.1172/jci.insight.164534

**Published:** 2022-12-08

**Authors:** Mou Wen, Yuanlin Ying, Xiang Xiao, Preston R. Arnold, Guangchuan Wang, Xiufeng Chu, Rafik M. Ghobrial, Xian C. Li

**Affiliations:** 1 Immunobiology and Transplant Science Center and Department of Surgery, Houston Methodist Hospital, Texas Medical Center, Houston, Texas, USA.; 2 Department of Thoracic Surgery, the Second Xiangya Hospital, Central South University, Changsha, China.; 3 Department of Surgery, Weill Cornell Medical College of Cornell University, New York, New York, USA.

**Keywords:** Autoimmunity, Immunology, Adaptive immunity, T cells

## Abstract

BRD4 is a bromodomain extraterminal domain family member and functions primarily as a chromatin reader regulating genes involved in cell-fate decisions. Here, we bred *Brd4^fl/fl^ Ox40-Cre* mice in which *Brd4* was conditionally deleted in OX40-expressing cells to examine the role of BRD4 in regulating immune responses. We found that the *Brd4^fl/fl^ Ox40-Cre* mice developed profound alopecia and dermatitis, while other organs and tissues were not affected. Surprisingly, lineage-tracing experiments using the *Rosa26^fl/fl^-Yfp* mice identified a subset of hair follicle stem cells (HFSCs) that constitutively express OX40, and deletion of *Brd4* specifically in such HFSCs resulted in cell death and a complete loss of skin hair growth. We also found that death of HFSCs triggered massive activation of the intradermal γδ T cells, which induced epidermal hyperplasia and dermatitis by producing the inflammatory cytokine IL-17. Interestingly, deletion of *Brd4* in Foxp3^+^ Tregs, which also constitutively express OX40, compromised their suppressive functions, and this, in turn, contributed to the enhanced activation of γδ T cells, as well as the severity of dermatitis and hair follicle destruction. Thus, our data demonstrate an unexpected role of BRD4 in regulating skin follicle stem cells and skin inflammation.

## Graphical abstract

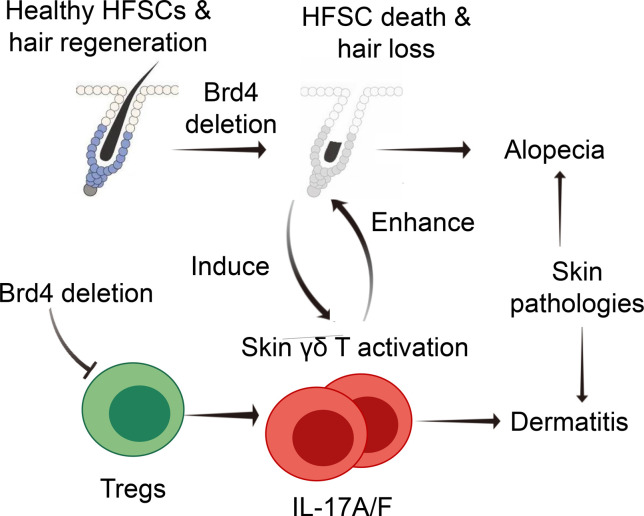

## Introduction

Skin diseases include all conditions that disrupt normal skin structure, integrity, and homeostatic responses, and the immune cells, including both inflammatory cells and T cells, are often closely involved (1). Among the diverse signals that control T cell activities, those from the T cell costimulatory molecules play a significant role (2). OX40 is a costimulatory molecule in the TNF receptor superfamily primarily involved in Treg proliferation, survival, and memory generation (3, 4). In naive mice, OX40 is mainly expressed by Foxp3^+^ Tregs (5). However, upon immune activation, activated T effector cells also express OX40, and upon binding its ligand on the APCs, OX40 signals exerts profound effects on the outcome of immune responses (6). For example, under certain conditions, OX40 signaling antagonizes Foxp3^+^ Tregs while boosting T effector responses, which provides an effective means of promoting antitumor immunity (7, 8). Moreover, in models of autoimmunity and transplant rejection, blocking OX40 signaling prevents activated T cells from acquiring effector and memory features, thus showing a remarkable therapeutic potential in those disease models (9, 10). Clearly, these findings suggest that OX40 can be a valuable therapeutic target in the clinic.

As a member in the TNF receptor superfamily, OX40 traditionally signals through the NF-κB pathway (11). We reported that, in freshly activated T cells, OX40 engagement strongly activates the noncanonical NF-κB pathway (i.e., RelB/p52) (12), thereby supporting the differentiation of Th9 cells and allergic inflammation at the costs of Tregs and Th17 cells. Interestingly, suppression of Th17 cells and Foxp3^+^ Tregs by OX40 is linked to profound chromatin remodeling at the *RORt* target genes, as well as at the *Foxp3* locus, where RelB triggers repressive chromatin modifications to close those gene loci, thus preventing the expression of key “master” transcription factors and, consequently, the differentiation of Th17 cells and Tregs (13). In Th9 cells, however, OX40 signaling results in the assembly of “super enhancers,” a large chromatin segment with exceptionally high levels of histone acetylation and transcription factor occupancy, to support Th9 induction (10). It has been reported that the super enhancers often regulate genes that are critical to cell-fate decisions and that the assembly of super enhancers relies on the bromodomain and extraterminal domain (BET) protein BRD4 (14, 15). In fact, BRD4 is a multidomain protein composed of 2 tandem bromodomains, an extraterminal domain, and a long C-terminal motif (16); both bromodomains contain a deep acetyl-lysine binding pocket, which allows BRD4 to selectively bind acetylated chromatin (i.e., H3K27ac) at activated gene enhancer and promoter sites (17). Thus, BRD4 acts as a chromatin reader, and by recruiting other chromatin modifiers, transcription factors, and co-factors to the enhancer sites, BRD4 drives the assembly of super enhancers that mediate robust transcription of target genes (18). In our studies, we found that OX40 is remarkably efficient at driving super enhancer assembly in Th9 cells via the noncanonical NF-κB pathway, and we found that inhibition of BRD4 using the chemical inhibitor JQ1 can effectively dismantle such super enhancers and consequently blocks the induction of Th9 cells (10, 12).

Given the diverse effects of OX40 in regulating T cell fates, including the generation of long-term memory and the finding that OX40 is also capable of activating other signaling pathway (e.g., PI3K/AKT) (19), it remains unclear as to what extent OX40 exerts its effects via BRD4-mediated chromatin remodeling. In the present study, we addressed this question by generating a potentially novel animal model, in which *Brd4* is selectively deleted in OX40-expressing cells using the *Ox40-Cre* recombinase. We found that the *Brd4^fl/fl^ Ox40-Cre* mice spontaneously develop profound alopecia and skin inflammation. Through a series of experiments involving T cell transfer, generation of BM chimera, and breeding to immune deficient *Rag1^–/–^* mice, we demonstrated that the alopecia and skin inflammation are mediated by different mechanisms. Specifically, we identified a subset of HFSCs that constitutively express OX40 and BRD4, and deletion of *Brd4* resulted in death of follicle stem cells, which then triggered the activation of γδ T cells. The activated γδ T cells then produced high levels of IL-17 and pronounced dermatitis. Thus, our results reveal a role for the chromatin reader BRD4 in regulating the HFSCs and skin inflammation.

## Results

### Conditional deletion of Brd4 in OX40-expressing cells induces prominent alopecia and severe dermatitis.

**Figure 1:**
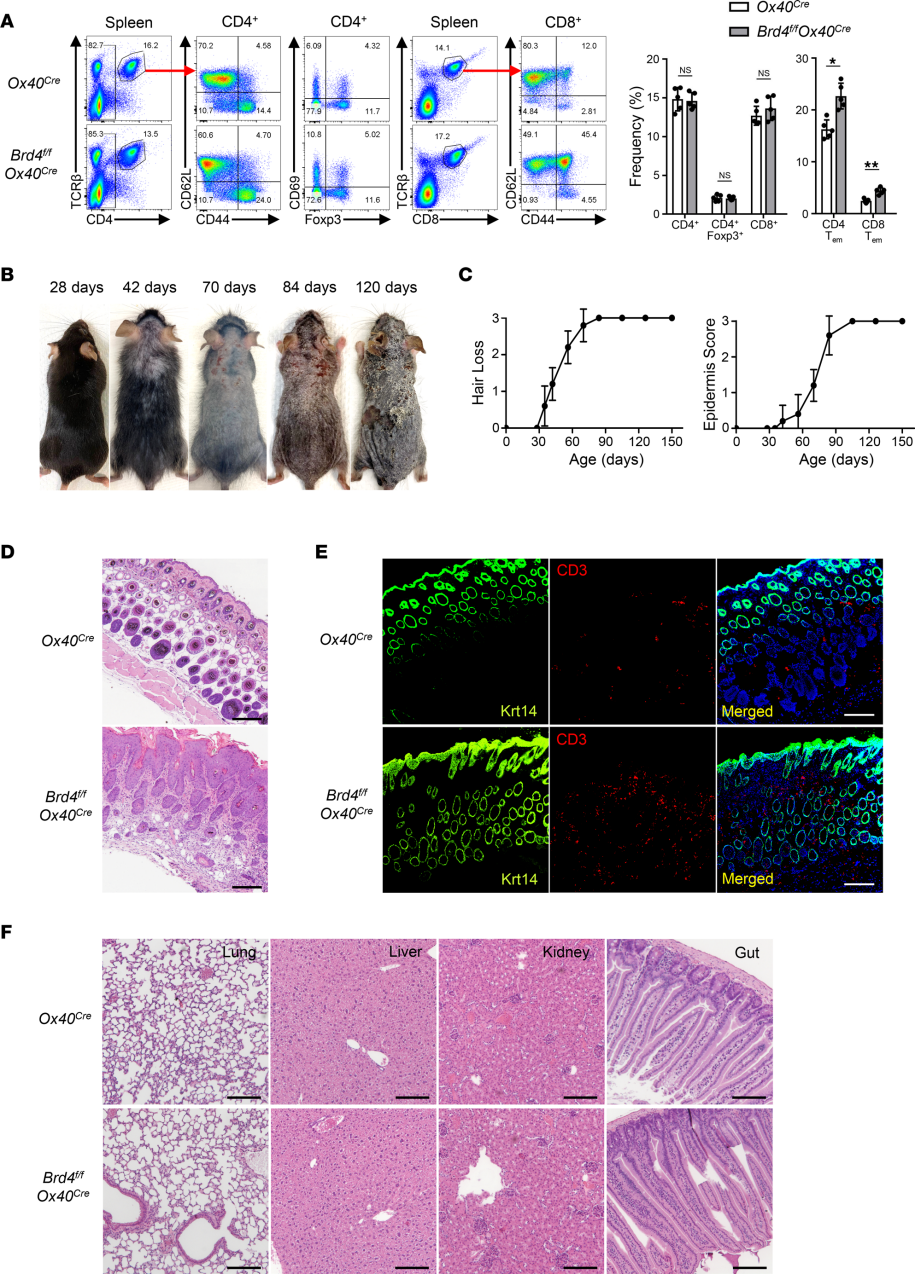
Deletion of *Brd4* using the *Ox40-Cre* recombinase results in progressive skin diseases. (**A**) Flow cytometric analysis of spleen CD4^+^, CD8^+^, Foxp3^+^, and memory T cells from 5-week-old *Ox40-Cre* and *Brd4^fl/fl^ Ox40-Cre* mice (left) and compiled data of all experiments in histograms (right). *n* = 5. (**B**) Gross appearance of dorsal skin of *Brd4^fl/fl^ Ox40-Cre* mice on P28, P42, P70, P84, and P120, showing progressive skin pathologies. (**C**) The relative score of hair loss (left) and epidermis score (right) calculated from *Brd4^fl/fl^ Ox40-Cre* mice over time. *n* = 5–7. (**D**) H&E staining of skin sections from *Ox40-Cre* and *Brd4^fl/fl^ Ox40-Cre* mice. Scale bar: 150 μm. (**E**) Immunofluorescence staining of keratin 14 (green) and CD3 (red) together with DAPI (third column) in dorsal skin sections from *Ox40-Cre* and *Brd4^fl/fl^ Ox40-Cre* mice. Scale bar: 120 μm. (**F**) H&E staining of tissue sections from *Ox40-Cre* and *Brd4^fl/fl^ Ox40-Cre* mice as indicated. Scale bar: 150 μm. Mice were at the age of 84 days (**D**–**F**). Data shown are representative results from 3 experiments (**A** and **D**–**F**) and 5 experiments (**B**). Graphs are shown as mean ± SD. *P < 0.05, **P < 0.01, by 2-tailed Student’s *t* test (**A**).

To determine the role of BRD4 in OX40-mediated effects, we crossed the *Brd4^fl/fl^* mice with *Ox40-Cre* mice, and *Brd4* was selectively deleted in OX40-expressing cells. We found that the *Brd4^fl/fl^ Ox40-Cre* mice appeared normal and healthy before 5 weeks of age, and a detailed analysis of their lymphoid compartments did not reveal marked abnormalities. As shown in Figure 1A, the *Brd4^fl/fl^ Ox40-Cre* mice had comparable CD4^+^, CD8^+^, and Foxp3^+^ Tregs in the spleen as compared with the *Ox40-Cre* controls. Similar findings were also observed in the lymph nodes (data not shown). The *Brd4^fl/fl^ Ox40-Cre* mice had an increased frequency of T cells with an effector memory phenotype (i.e., CD44^hi^CD62L^–^), but this increase was not remarkable at 5 weeks of age (Figure 1A). Interestingly, the *Brd4^fl/fl^ Ox40-Cre* mice developed alopecia starting at 6 weeks age (Figure 1, B and C), and by the time they reached 10 weeks of age, dermatitis then developed, which became worse over time and often necessitated euthanasia (Figure 1, B and C). None of those changes were observed in the control *Ox40-Cre* or *Brd4^fl/fl^* mice. Tissue histology of the skin from *Brd4^fl/fl^ Ox40-Cre* mice at 12 weeks of age revealed marked epidermal hyperplasia, hyperkeratosis, loss of hair follicles, and inflammatory cell infiltration in the skin, which contrasted sharply with the skin histology of age-matched control mice (Figure 1D). Moreover, immunofluorescence staining of the inflamed skin confirmed the accumulation of CD3^+^ T cells in the dermal layer of *Brd4^fl/fl^ Ox40-Cre* mice, as well as hyperplasia of epidermis (Figure 1E). We also examined other organs from the *Brd4^fl/fl^ Ox40-Cre* mice for signs of inflammation, including the lung, liver, kidney, and gut. Surprisingly, we failed to find any pathological changes at those sites, and their histological features were essentially identical between *Brd4^fl/fl^ Ox40-Cre* mice and the control mice (Figure 1F), suggesting that the pathological changes in *Brd4^fl/fl^ Ox40-Cre* mice are confined to the skin.

### Lineage-tracing studies identify OX40 expression by HFSCs.

**Figure 2:**
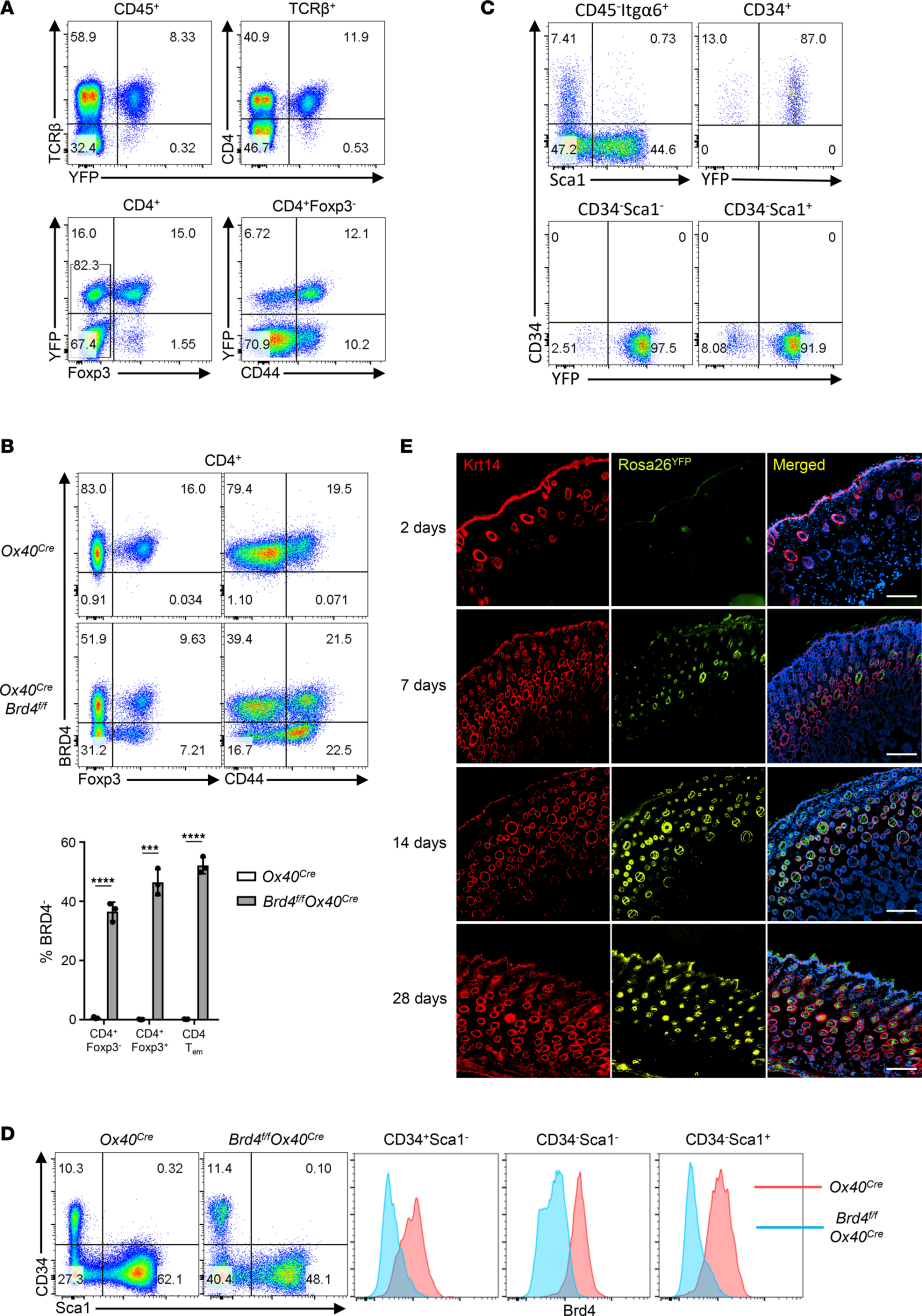
Genetic lineage tracing of OX40-expressing cells in the immune system and skin parenchyma. (**A**) Flow cytometric analysis of *Rosa26-YFP*^+^ cells in peripheral lymph nodes from *Rosa26^fl/fl^-Yfp*–*Ox40-Cre* mice gated on lymphoid lineage (CD45^+^) live cells. *n* = 5. (**B**) Flow cytometric analysis of intracellular BRD4 expression in CD4^+^ cells gated on TCR-β^+^ cells in lymph nodes of *Ox40-Cre* and *Brd4^fl/fl^ Ox40-Cre* mice (top), and the percentage of BRD4^–^ cells in the bar graph (bottom). *n* = 3. (**C**) Flow cytometric analysis of *Rosa26-YFP*^+^ skin parenchymal cells (CD45^–^Itgα6^+^) isolated from dorsal skin of *Rosa26^fl/fl^-Yfp*–*Ox40-Cre* mice. HFSCs were marked as CD45^–^Itgα6^+^CD34^+^Sca1^–^. *n* = 5. (**D**) Intracellular expression of BRD4 in follicle stem cells gated onto the CD45^–^Itgα6^+^ live cells isolated from dorsal skin of *Ox40-Cre* and *Brd4^fl/fl^ Ox40-Cre* mice. *n* = 3. Mice were at the age of 8 weeks (**A**–**D**). (**E**) Immunofluorescence of keratin 14 (red) and YFP (green), and both merged with DAPI staining (third column) in dorsal skin sections of *Rosa26^fl/fl^-Yfp*–*Ox40-Cre* mice at the indicated ages. Data shown in all panels are representative results from 3 experiments. Graphs are shown as mean ± SD. ***P < 0.001, ****P < 0.0001, by 2-tailed Student’s *t* test (**B**).

To dissect whether alopecia and dermatitis are manifestations of the same disease or involve different mechanisms, we firstly bred the *Rosa26^fl/fl^-Yfp* mice with *Ox40-Cre* mice to generate *Rosa26^fl/fl^-Yfp*–*Ox40-Cre* mice, in which cells that have ever-expressed OX40 (past and present) are genetically marked by the yellow fluorescent protein (YFP). As shown in Figure 2A, OX40 was almost exclusively expressed by T cells in the immune system (i.e., TCR-β^+^CD45^+^ cells), in which ~96% of the YFP^+^ T cells were also CD4^+^ (Figure 2A). Within the CD4^+^ subset, however, ~50% of the YFP^+^ cells also expressed Foxp3, and among CD4^+^Foxp3^+^ Tregs, an overwhelming majority expressed YFP (>90%). In the CD4^+^YFP^+^Foxp3^–^ subset, most of the T cells were CD44^hi^, suggesting a memory phenotype of those T cells. Thus, in naive mice, OX40 is primarily expressed by Foxp3^+^ Tregs and a subset of CD4^+^ memory cells (Figure 2A), which is consistent with previous reports (20). Interestingly, virtually all CD4^+^Foxp3^+^ Tregs expressed BRD4 in control *Ox40-Cre* mice, and ~60% of the Foxp3^+^ Tregs in *Brd4^fl/fl^ Ox40-Cre* mice lost BRD4 expression (Figure 2B), which is in line with the expression of OX40 by Tregs (Figure 2A).

We also traced the skin parenchymal cells for YFP expression in an attempt to understand why the tissue pathology is confined to the skin. Surprisingly, we found substantial *Ox40-Cre* activities and consequently YFP expression in the skin (Figure 2C). In fact, YFP was expressed at high levels in CD34^+^ follicle stem cells, as well as in cell types derived from such stem cells, which include the epidermal cells, hair germ cells, and sheath cells (both CD34^–^Sca1^+^ and CD34^–^Sca1^–^ subsets) (21). As compared with *Ox40-Cre* control mice, all subsets of those skin cells, which constitutively express BRD4, showed markedly reduced expression of BRD4 in the *Brd4^fl/fl^ Ox40-Cre* mice (Figure 2D). Moreover, immunofluorescence staining of the skin, using keratin 14 staining as profiles of hair follicles and epidermis, showed that the YFP was abundantly expressed in the hair follicles and epidermis across a wide range of time points examined (7–28 days of age) (Figure 2E), thus further supporting the expression of OX40 by follicle stem cells. Together, these findings suggest that, in the *Brd4^fl/fl^ Ox40-Cre* mice, *Brd4* was conditionally deleted in both immune cells and HFSCs.

### Deletion of Brd4 in T cells is not sufficient to induce skin pathology.

**Figure 3:**
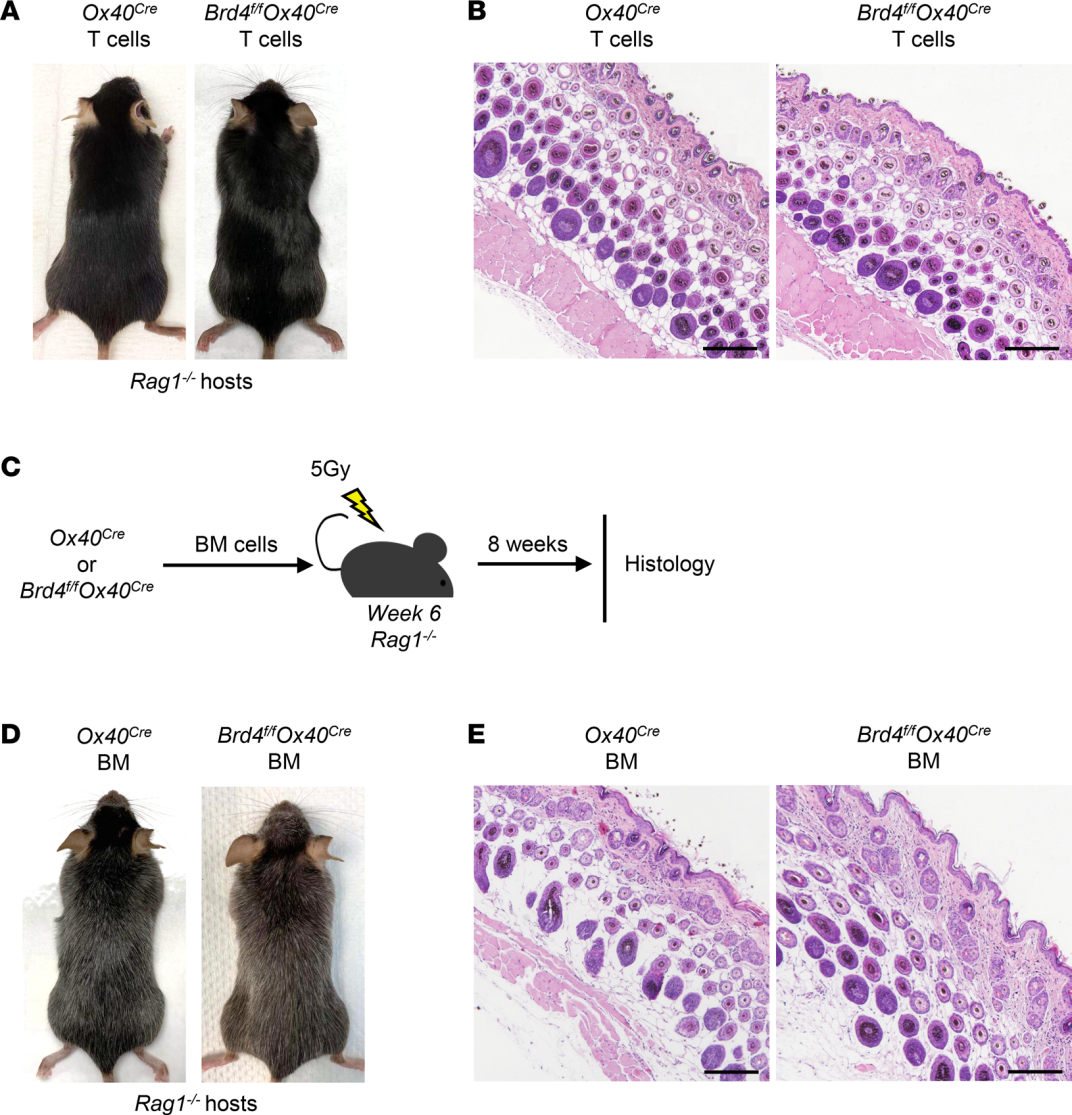
Deletion of *Brd4* in T cells or hematopoietic stem cells alone is not sufficient to trigger skin pathology. (**A**) Gross appearance of 4-week-old *Rag1^–/–^* recipients 6 weeks after transfer of T cells from *Ox40-Cre* and *Brd4^fl/fl^ Ox40-Cre* mice. (**B**) Skin histology (H&E staining) of *Rag1^–/–^* mice injected with *Ox40-Cre* and *Brd4^fl/fl^ Ox40-Cre* T cells. Scale bar: 150 μm. (**C**) Schematic diagram of BM chimeric procedure in 6-week-old *Rag1^–/–^* recipients. (**D**) Gross appearance of *Rag1^–/–^* recipients 6 weeks after reconstitution with BM cells from *Ox40-Cre* and *Brd4^fl/fl^ Ox40-Cre* mice. (**E**) Skin sections (H&E staining) of recipient mice reconstituted with *Ox40-Cre* and *Brd4^fl/fl^ Ox40-Cre* BM cells. Scale bar: 150 μm. The data are representative of at least 3 independent experiments, each with *n* = 4–6.

We took several approaches to determine the role of *Brd4*-deleted T cells in skin pathology. Firstly, we adoptively transferred purified T cells from the control *Ox40-Cre* mice and *Brd4^fl/fl^ Ox40-Cre* mice (8 weeks of age) into the syngeneic *Rag1^–/–^* hosts. The host mice were followed for signs of skin diseases. As shown in Figure 3A, the *Rag1^–/–^* hosts in both groups remained healthy, with thick hair coat and no skin lesions observed 6–10 weeks after T cell transfer. The H&E staining of skin sections from both groups showed an intact epidermis, normal hair follicle structures, and a complete absence of inflammatory cell infiltrates (Figure 3B). These results indicate that deletion of *Brd4* in T cells alone is not sufficient to induce hair loss and skin inflammation. We secondly generated BM chimeric mice, where the BM cells from *Brd4^fl/fl^ Ox40-Cre* mice and control *Ox40-Cre* mice were used to reconstitute the lethally irradiated *Rag1^–/–^* mice (Figure 3C). Six to 8 weeks after BM reconstitution, the host mice were examined for skin diseases. As shown in Figure 3D, the host *Rag1^–/–^* mice were grossly normal with no overt hair loss and dermatitis, regardless of reconstitution with control or *Brd4^fl/fl^ Ox40-Cre* BM cells. Again, the skin histology showed well-preserved hair follicles with no epidermal hyperplasia and inflammatory cell infiltration (Figure 3E). Thus, selective deletion of *Brd4* in T cells or in hematopoietic cells alone does not seem to be the underlying mechanism of skin diseases observed in the *Brd4^fl/fl^ Ox40-Cre* mice.

### Deletion of Brd4 in follicle stem cells disrupts follicle structures and blocks hair regeneration.

**Figure 4:**
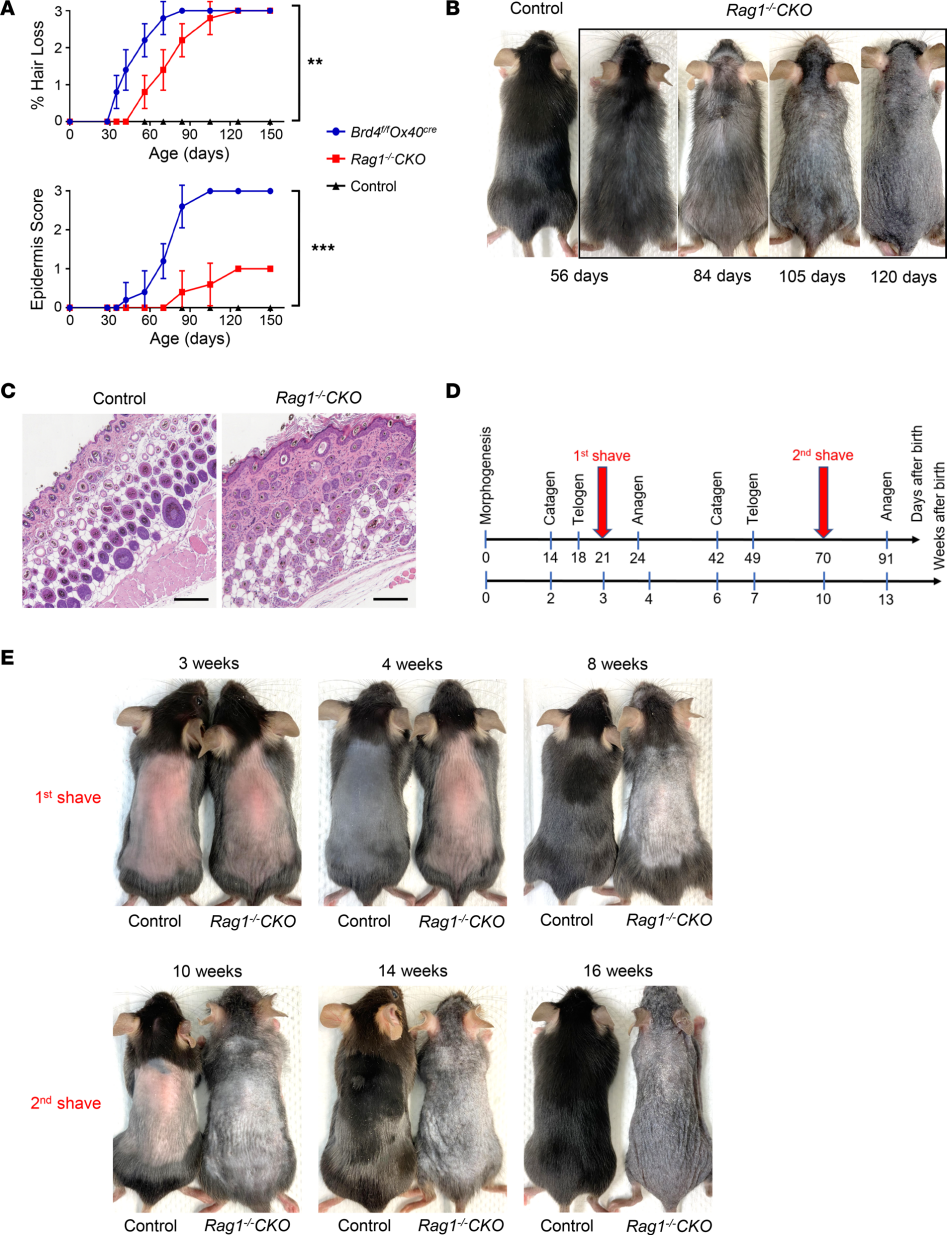
Effect of *Brd4* deletion specifically in HFSCs on hair growth and skin diseases. (**A**) Average percentage of hair loss (top) and epidermis score (bottom) for *Brd4^fl/fl^ Ox40-Cre*, *Rag1^–/–^ Brd4^fl/fl^ Ox40-Cre* (*Rag1^–/–^* CKO), and *Rag1^–/–^ Ox40-Cre* (control) mice over time. *n* = 5–7. (**B**) Gross appearance of skin hair in *Rag1^–/–^* CKO mice over time as compared with control mice at 8 weeks old. (**C**) Skin sections (H&E staining) of 12-week-old *Rag1^–/–^* CKO and control mice. Scale bar: 150 μm. (**D**) Schematic diagram showing dorsal skin hair shaving points in *Rag1^–/–^* CKO and control mice during telogen stages. (**E**) *Rag1^–/–^* CKO and control mice were monitored for hair regeneration after hair shaving. Photographs show dorsal hair conditions at indicated time points. Data shown in all panels are representative results from 3 experiments. Graphs are shown as mean ± SD. **P < 0.01, ***P < 0.001, by 1-way ANOVA (**A**).

To further dissect the underlying mechanisms of skin pathology, we bred *Brd4^fl/fl^ Ox40-Cre* mice into the *Rag1^–/–^* background (*Rag1^–/–^ Brd4^fl/fl^ Ox40-Cre* [*Rag1^–/–^* CKO]), where deletion of *Brd4* was confined to HFSCs, as well as cells derived from them. We observed that when compared with the control (*Rag1^–/–^ Ox40-Cre*) mice, the *Rag1^–/–^* CKO mice showed thinning of hair density starting at 56 days of age (Figure 4A), and by the time they reached 100 days of age, the *Rag1^–/–^* CKO mice lost almost all their hair (Figure 4, A and B). However, as compared with the *Brd4^fl/fl^ Ox40-Cre* mice, the hair loss in *Rag1^–/–^* CKO mice was delayed and at a slower rate, and dermatitis was absent throughout the observation time (Figure 4, A and B). We also performed H&E staining on the skin tissues on P84, and as shown in Figure 4C, the skin from *Rag1^–/–^* CKO mice revealed a thickened epidermis and markedly disorganized follicle structures, which contrasted with the appearance of the control mice. In fact, the hair follicle structure was largely collapsed in the *Rag1^–/–^* CKO mice (Figure 4C), demonstrating a profound effect of *Brd4* deletion on skin hair follicles.

As highlighted in Figure 4D, hair follicles undergo cyclic bouts of catagen (degeneration), telogen (quiescence), and anagen (regeneration) to maintain hair growth and regeneration (22–24), and the most robust cell proliferation in the hair cycle occurs within days around telogen-anagen transition (25, 26). To determine whether the hair loss in Rag1^–/–^ CKO mice is due to a failure of telogen-anagen transition, we physically shaved the mice twice, once on day 21 right before the first anagen phase and again on day 70, which was before the second anagen phase (Figure 4D). We then monitored hair growth in the shaved Rag1^–/–^ Ox40-Cre control and Rag1^–/–^ CKO mice. As shown in Figure 4E, the hair readily grew back in the dorsal skin of control mice after the first and the second shaves, forming a dark and healthy skin hair coat. In stark contrast, the shaved Rag1^–/–^ CKO mice completely failed to regenerate their skin hair after both shaves. Additionally, skin thickening and acanthosis-like appearance were observed in the shaved Rag1^–/–^ CKO mice at 14 weeks of age (Figure 4E). These data suggest that deletion of Brd4 in HFSCs arrests them at the telogen phase, preventing them from entering the hair cycle during hair regeneration.

### Brd4-deleted HFSCs fail to proliferate and readily die in situ.

**Figure 5:**
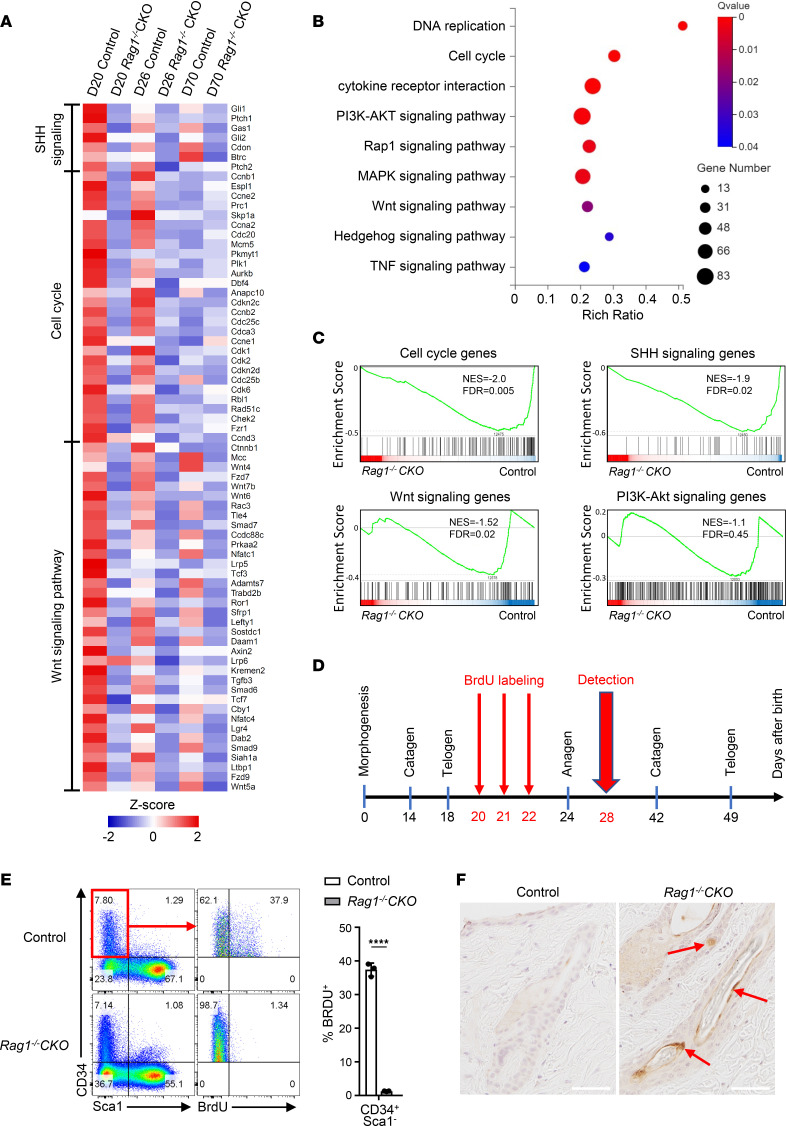
Gene transcriptional profiles, survival, and proliferation of HFSCs with or without *Brd4* deletion. (**A**) Heatmap plot of RNA-Seq data showing expression of SHH signaling genes, cell cycle–related genes, and Wnt signaling pathway genes in follicle stem cells of *Rag1^–/–^* CKO and control mice at different ages as indicated. Positive *Z* score depicts higher expression; negative *Z* score indicates lower expression. (**B**) KEGG pathway enrichment analysis of differentially expressed genes in *Rag1^–/–^* CKO and control follicle stem cells on P20. (**C**) Gene set enrichment analysis of differentially expressed genes in the signaling pathways as indicated between *Rag1^–/–^* CKO and control HFSCs on P20. (**D**) Schematic diagram showing BrdU labeling and detection time points for *Rag1^–/–^* CKO and control follicle stem cells. (**E**) Flow cytometry analysis of BrdU uptake in *Rag1^–/–^* CKO and control follicle stem cells (left) and frequency of BrdU^+^ cells in CD34^+^Sca1^–^ subset (right). *n* = 3. (**F**) TUNEL assay for apoptotic death of follicle stem cells in skin sections from *Rag1^–/–^* CKO and control mice. Photographs show hair follicles. Arrows show cell death. Scale bar: 60 μm. Data shown are representative results from 4 experiments (**E**) and 3 experiments (**F**). Graphs are shown as mean ± SD. ****P < 0.0001, by 2-tailed Student’s *t* test (**E**).

To identify the molecular mechanisms of impaired hair regeneration, we sorted HFSCs from *Rag1^–/–^ Ox40-Cre* and *Rag1^–/–^* CKO mice by FACS and performed RNA-Seq analysis comparing differences in their transcriptional profiles. As shown in Figure 5A, *Brd4* deletion led to broad transcriptional changes in follicle stem cells, especially on P20, a time point when follicle stem cells are mostly active in preparation for anagen entry (25, 26). Interestingly, genes that showed the most striking differences between control and *Brd4*-deleted follicle stem cells were related to the sonic hedgehog (SHH) signaling, cell cycle, and Wnt signaling pathways, where *Brd4* deletion led to their marked downregulation. Similar downregulation of those genes was also observed on day 26 when follicle stem cells are at the anagen phase. Such transcriptional differences were less striking, however, on day 70, when the follicle stem cells had already entered the telogen state for 3 weeks and, therefore, are quiescent (Figure 5A). Moreover, pathway analysis (Kyoto Encyclopedia of Genes and Genomes [KEGG]) revealed that the cell cycle, Wnt, SHH, and PI3K/AKT signaling pathways were markedly downregulated in *Brd4*-depleted stem cells (Figure 5B), and gene set enrichment analysis (GSEA) further confirmed that, in *Brd4*-deleted follicle stem cells, the downregulated genes were mostly confined to the cell cycle, SHH, and Wnt signaling pathways (Figure 5C). We then accessed the published sequencing database (27), identified super enhancer elements in follicle stem cells, and examined epigenetic features of genes in the above-mentioned signaling pathways, as well as lineage-specific transcription factor occupancy. We observed that, in normal follicle stem cells, there were enhanced H3K27ac ChIP-Seq peaks (Supplemental Figure 1A; supplemental material available online with this article; https://doi.org/10.1172/jci.insight.164534DS1). Quantitative PCR (qPCR) showed that, in *Brd4*-deleted follicle stem cells, genes critical to follicle stem cell activation and proliferation were dramatically downregulated (Supplemental Figure 1B).

To further assess the survival and proliferation of *Brd4-*deleted follicle stem cells in vivo, we treated the control and *Rag1^–/–^* CKO mice with BrdU, as depicted in Figure 5D, and examined BrdU incorporation into proliferating HFSCs. As shown in Figure 5E, follicle stem cells in control mice, marked as CD34^+^Sca1^–^, showed significant BrdU uptake (~38%), suggesting a state of active proliferation. In contrast, the stem cells from *Rag1^–/–^* CKO mice completely failed to uptake BrdU, indicating a state of quiescence (Figure 5E). Remarkably, staining for cell death using the TUNEL assay revealed increased follicle cell death in *Rag1^–/–^* CKO mice as compared with the controls (Figure 5F, red arrows). This finding helps explain the collapsed hair follicle structures observed in *Rag1^–/–^* CKO mice (Figure 4C). Together, these data identify a key role for BRD4 in HFSCs, especially during the telogen-anagen transition.

### γδ T cells mediate dermatitis via production of IL-17 and IFN-γ.

**Figure 6:**
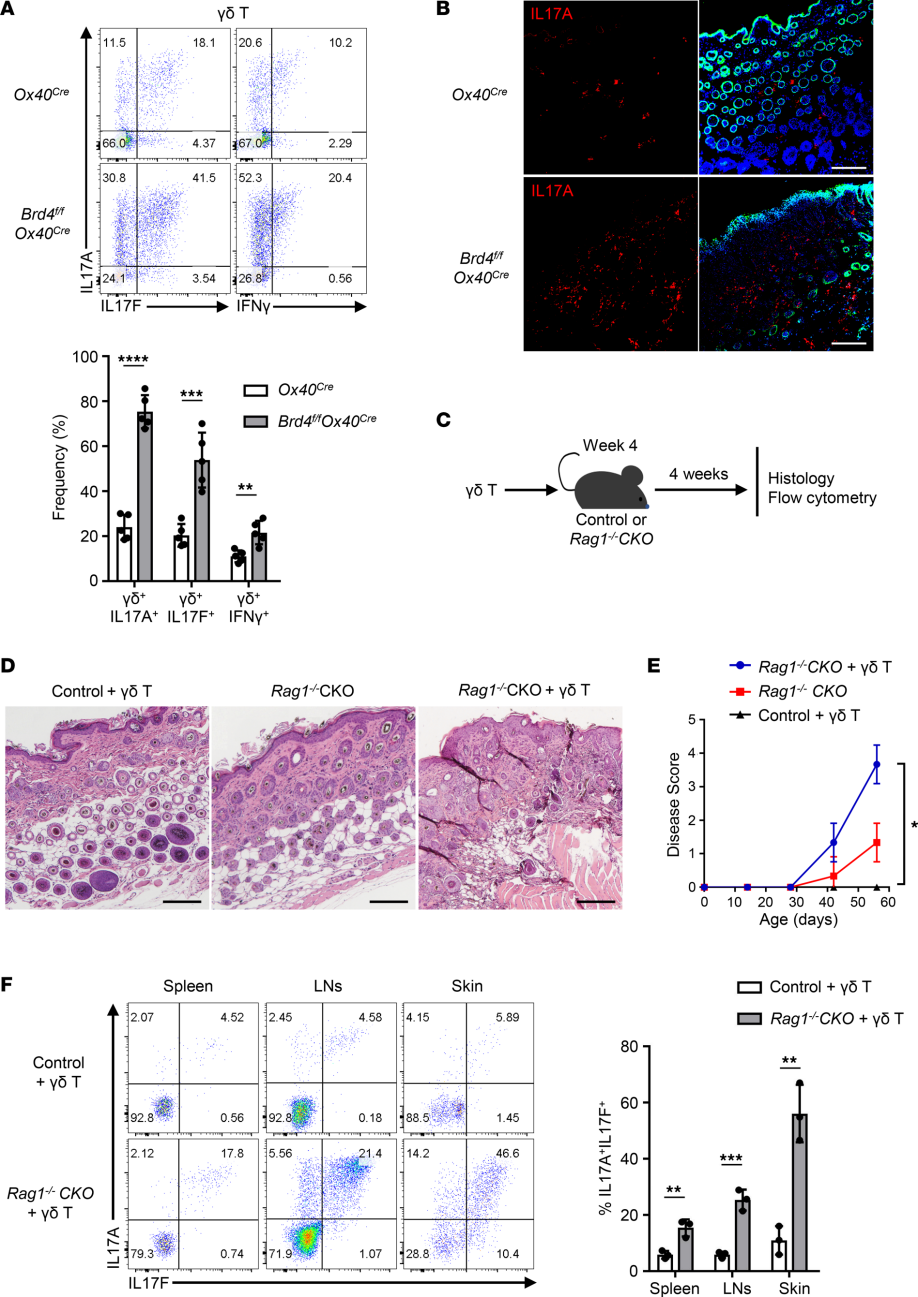
Activation of γδ T cells in dermatitis and hair follicle destruction. (**A**) Flow cytometry analysis of IL-17 and IFN-γ expression in γδ^+^ T cells from the skin of control *Ox40-Cre* and *Brd4^fl/fl^ Ox40-Cre* mice on P84 (top), and tabulated data from all mice as indicated (bottom). *n* = 5. (**B**) Immunofluorescence staining of IL-17A (red) stain together with DAPI (blue) and keratin 14 (green) in dorsal skin sections from control *Ox40-Cre* and *Brd4^fl/fl^ Ox40-Cre* mice on P84. Scale bar: 120 μm. (**C**) Schematic diagram showing γδ T cells transfer into *Rag1^–/–^* CKO and control recipients. (**D**) Skin sections (H&E staining) of *Rag1^–/–^* CKO mice, control plus γδ T, and *Rag1^–/–^* CKO plus γδ T recipients. Scale bar: 150 μm. (**E**) Graph shows average disease score for *Rag1^–/–^* CKO mice, control plus γδ T, and *Rag1^–/–^* CKO plus γδ T recipients over time. *n* = 5–7. (**F**) Flow cytometry analysis of intracellular expression of IL-17A and IL-17F in transferred γδ T cells isolated from spleen, lymph nodes, and skin of control plus γδ T and *Rag1^–/–^* CKO plus γδ T recipients (left) and expression levels of IL-17A and IL-17F expression in γδ T cells from all mice (right). *n* = 3. Data shown are representative results from 3 experiments (**A**, **D**, and **F**) and 4 experiments (**B**). Graphs shown as mean ± SD. *P < 0.05, **P < 0.01, ***P < 0.001, ****P < 0.0001, by 1-way ANOVA (**E**) or 2-tailed Student’s *t* test (**A** and **F**).

Since the *Brd4^fl/fl^ Ox40-Cre* mice develop severe skin inflammation, whereas the *Rag1^–/–^* CKO mice exhibit alopecia without skin inflammation, we then examined the identity of T cells and to what extent they mediate the skin inflammation in *Brd4^fl/fl^ Ox40-Cre* mice. We firstly extracted lymphoid cells from the skin of *Ox40-Cre* and *Brd4^fl/fl^ Ox40-Cre* mice (12 weeks old) and analyzed them with flow cytometry by gating onto the CD4^+^ and γδ^+^ T cells (major skin resident subsets). We found that the γδ^+^ T cells did not express OX40 (Supplemental Figure 2A), but they had an activated phenotype in *Brd4^fl/fl^ Ox40-Cre* mice and produced elevated levels of IL-17 (both IL-17A and IL-17F) and IFN-γ as compared with those in the control *Ox40-Cre* mice (Figure 6A). The CD4^+^ T cells in the skin had a barely detectable IL-17 and IFN-γ production, regardless of *Brd4* deletion (Supplemental Figure 2B). Immunofluorescence staining of the skin tissue further confirmed the increased production of IL-17 in the epidermis of *Brd4^fl/fl^ Ox40-Cre* mice (Figure 6B), suggesting that γδ^+^ T cells are likely the effector cells in skin inflammation in conditional *Brd4*-deleted mice.

To further determine the pathological role of γδ^+^ T cells in our model, we performed a series of adoptive cell transfer experiments, where the γδ^+^ T cells sorted by FACS from WT B6 mice were adoptively transferred into *Rag1^–/–^ Ox40-Cre* controls and *Rag1^–/–^* CKO recipients, and the recipient mice were evaluated for skin pathologies (Figure 6C). As shown in Figure 6D, the *Rag1^–/–^* CKO mice that received the γδ^+^ T cells developed prominent skin inflammation, characterized by inflammatory cell infiltrates, epidermal hyperplasia, and follicle destruction, while the control recipients displayed normal skin structures with well-organized hair follicles regardless of γδ^+^ T cell transfer. When the clinical scores were tabulated during the 4-week study period, the *Rag1^–/–^* CKO mice transferred with γδ^+^ T cells showed far more severe skin diseases as compared with the control mice (Figure 6E). Moreover, intracellular staining for IL-17 expression by the transferred γδ^+^ T cells showed that γδ^+^ T cells in the *Rag1^–/–^* CKO mice produced much higher levels of IL-17 than those in the *Rag1^–/–^ Ox40-Cre* control mice (Figure 6F). These results suggest that death of follicle stem cells in *Brd4^fl/fl^ Ox40-Cre* mice drives activation of the skin γδ^+^ T cells and their enhanced production of IL-17, together mediating the skin pathologies.

### The impaired Tregs in Brd4^fl/fl^ Ox40-Cre mice further contribute to skin inflammation.

**Figure 7:**
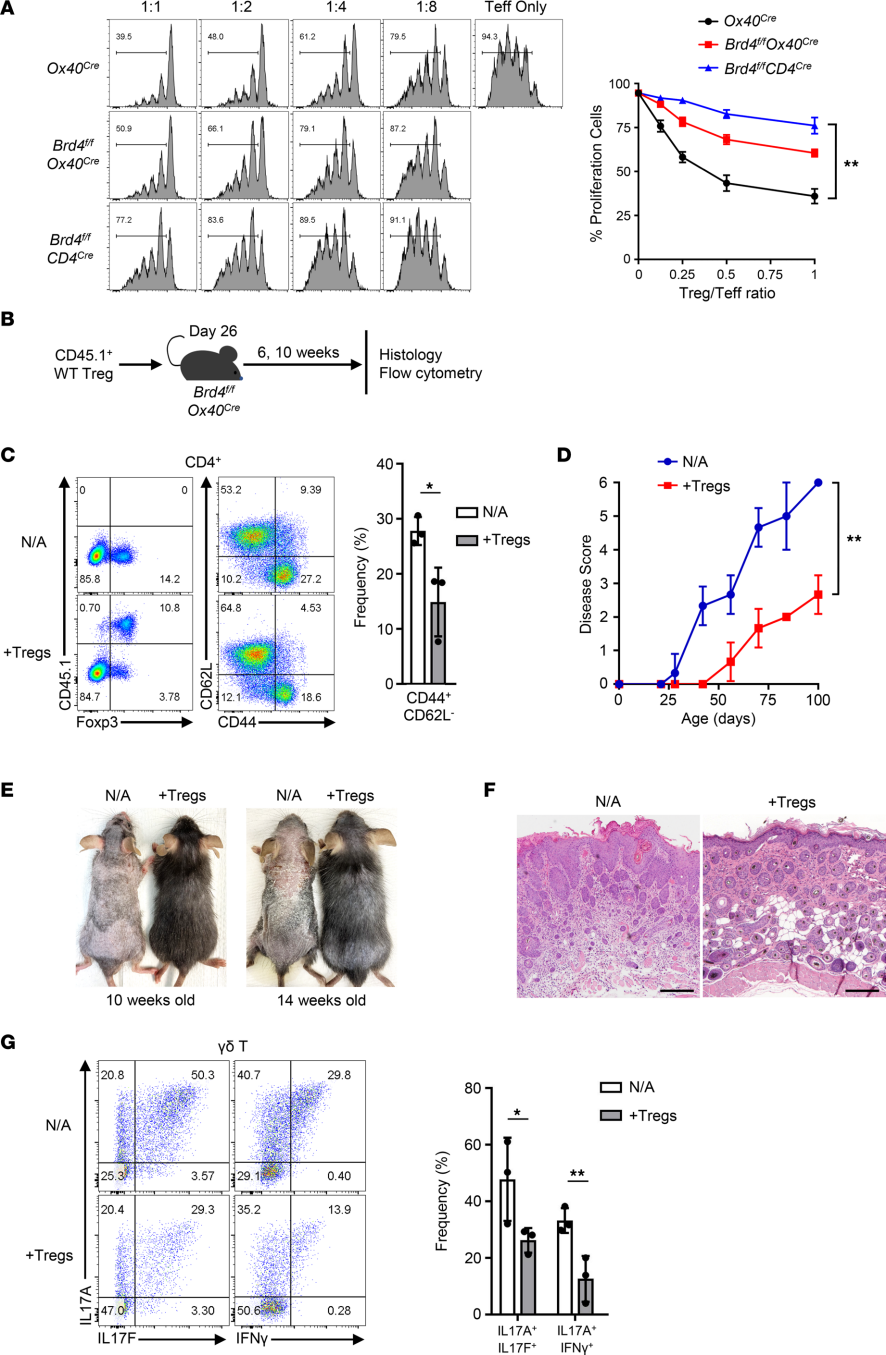
*Brd4* deletion in Tregs impairs their suppressive functions contributing to γδ T cell activation and skin inflammation. (**A**) In vitro suppression assays based on CFSE dilutions comparing suppressive functions of Foxp3^+^ Tregs from control *Ox40-Cre*, *Brd4^fl/fl^ Ox40-Cre*, and *Brd4^fl/fl^Cd4-Cre* mice. The ratio between Tregs and T effectors are indicated (left). The percentage of proliferating responder T cells at different T effector/Treg ratios is shown in the histogram (right). *n = 3*. (**B**) Schematic diagram showing adoptive transfer of WT Tregs (CD45.1^+^) to *Brd4^fl/fl^ Ox40-Cre* recipients. (**C**) Flow cytometric analysis of Foxp3^+^ Tregs and T effectors gated onto CD4^+^ cells from peripheral lymph nodes of CD45.1^+^ Treg recipients compared with nontransferred (N/A) mice (14 weeks old) (left) and frequency of CD44^+^CD62L^–^ cells in the recipients (right). *n* = 3. (**D**) Graph shows average disease score for CD45.1^+^ Treg recipients compared with N/A mice over time. *n* = 5–7. (**E**) Gross appearance of dorsal skin of CD45.1^+^ Treg recipients compared with N/A mice at 10 weeks and 14 weeks age. (**F**) Skin sections (H&E staining) of N/A mice and CD45.1^+^ Treg recipients. Scale bar: 150 μm. (**G**) Flow cytometry analysis of intracellular expression of cytokines (IL-17A, IL-17F, and IFN-γ) in γδ T cells isolated from skin of CD45.1^+^ Treg recipients compared with N/A mice at 14 weeks old (left), and frequency of IL-17A^+^IL-17F^+^ or IL-17A^+^IFN-γ^+^ cells (right). *n* = 3. Data shown are representative results from 2 experiments (**A**) and 3 experiments (**C** and **E**–**G**). Graphs shown as mean ± SD. *P < 0.05, **P < 0.01, by 1-way ANOVA (**A**) or 2-tailed Student’s *t* test (**C**, **D**, and **G**).

Since most Foxp3^+^ Tregs express OX40 and BRD4 under homeostatic conditions, we further examined whether the conditional *Brd4* deletion in Tregs would affect their functions and how that would contribute to the disease process in *Brd4^fl/fl^ Ox40-Cre* mice. As shown in Figure 7A, in an in vitro suppression assay, Foxp3^+^ Tregs from the *Brd4^fl/fl^ Ox40-Cre* mice were inferior at suppressing T effector cells as compared with those from the *Ox40-Cre* control mice. We observed a further impairment of Foxp3^+^ Tregs from *Brd4^fl/fl^Cd4-Cre* mice in terms of their suppressive functions. We also performed adoptive Treg transfer assay, where Tregs from control mice (CD45.1^+^) were transferred into *Brd4^fl/fl^ Ox40-Cre* mice (CD45.2^+^) on P26, and the host mice were then followed for 6–10 weeks after Treg transfer (Figure 7B). As shown in Figure 7C, the transferred CD45.1^+^ Tregs survived long-term in the *Brd4^fl/fl^ Ox40-Cre* recipients and maintained strong Foxp3 expression, whereas the host Tregs (*Brd4*-deleted Tregs) showed markedly reduced Foxp3 levels. Accordingly, CD4^+^ effector memory cells declined in frequency upon transferring functional Tregs (Figure 7C). As a result, the clinical signs of dermatitis were significantly improved in Treg-transferred *Brd4^fl/fl^ Ox40-Cre* mice as compared with those without Treg transfer (Figure 7D). Notably, at 10 weeks of age, the *Brd4^fl/fl^ Ox40-Cre* mice that received CD45.1^+^ Tregs had markedly reduced dermatitis and hair loss; similar findings were observed at 14 weeks of age, which contrasted sharply with those without Treg transfer (Figure 7E). Histologically, skin tissues in Treg-transferred hosts revealed reduced epidermal hyperplasia and reduced inflammatory cell accumulation, as well as better-organized hair follicles, as compared with *Brd4^fl/fl^ Ox40-Cre* mice without Treg transfer (Figure 7F). Furthermore, adoptive transfer of Tregs also suppressed the activation of γδ^+^ T cells and their production of the inflammatory cytokines IL-17 and IFN-γ (Figure 7G), and such a suppression of inflammatory cytokines was also observed in the host spleen and lymph nodes (Supplemental Figure 2, C and D). Collectively, these results suggest that deletion of *Brd4* in Tregs (*Ox40-Cre* mediated) weakens their regulatory functions, which contributes to γδ T cell activation and skin inflammation.

## Discussion

In the present study, we demonstrate that conditional deletion of *Brd4* using the *Ox40-Cre* recombinase induces tissue-specific skin inflammation characterized by profound alopecia and dermatitis. We provided strong evidence that the *Ox40-Cre* activities are present not only in the immune cells, but also in the HFSCs (nonimmune cells) and that the development of alopecia and dermatitis is mediated by very different mechanisms. Conditional deletion of *Brd4* in hair follicle stem cells inhibited their proliferation during hair growth cycles, which constitutes the underlying mechanisms of alopecia. Additionally, deletion of *Brd4* in follicle stem cells also rendered them sensitive to cell death, which in turn triggered the activation of γδ T cells and production of potent inflammatory cytokines, including IL-17A, IL-17F, and IFN-γ, that subsequently mediate skin inflammation. We also found that conditional deletion of *Brd4* in Foxp3^+^ Tregs, which constitutively express OX40 (7), impaired their suppressive functions both in vitro and in vivo, and this Treg defect further contributed to the enhanced activation of γδ T cells in the skin and, consequently, severe dermatitis. Thus, our data reveal potentially novel insights into the roles of BRD4 in the control of both immune cells and nonimmune cells in tissue-specific skin inflammation.

OX40 as a T cell costimulatory molecule involved in regulating T cell responses has been extensively studied in both clinical and preclinical models (28). The finding that OX40 signaling also regulates HFSCs via the chromatin reader BRD4 is highly interesting. In our lineage-tracing studies using the *Rosa26-Yfp* reporter mice, we provide strong evidence that the HFSCs constitutively express substantial *Ox40-Cre* activities as marked by the YFP expression (Figure 2). Others have also reported that deletion of either Pdk1 kinase or IkB kinase-2 using the same Ox40-Cre results in impaired skin epidermis and keratinocytes (29, 30), thus further supporting the importance of OX40 activities in skin homeostasis. However, the identification of BRD4 under the control of OX40 signaling in regulation of HFSCs is novel, to our knowledge. The hair growth cycle is tightly regulated under physiological conditions, where the follicle stem cells, which normally reside at the bulge region of hair follicles (22, 23), transition through distinct phases of activation (31), and through interactions with adjacent cells including hair germ cells, they transition from being quiescent in the telogen phase to becoming activated in anagen phase and then fuel hair regeneration (25, 26). It has been reported that the telogen-to-anagen transition involves the activation of Wnt/β-catenin/Lef1 and SHH signaling pathways, as well as cell cycle regulators (32–36). Indeed, our RNA-Seq analysis comparing transcriptional profiles between control and *Brd4*-deleted HFSCs shows that all those signaling pathways were strongly impacted by *Brd4* deletion (Figure 5). Previous studies using ChIP-Seq profiling demonstrate the importance of signal-dependent transcription factors in choreographing follicle stem cell lineages via chromatin modifications (37). We explored the published super enhancer database in follicle stem cells (27), and we found that some lineage transcription factors (*Lhx2*, *Nfib*, *Sox9*, *Dlx3*) and key signaling factors (*Lef1*, *Frfr2*, *Id1*, *Lgr4* for Wnt pathway; *Gas1*, *Hes1*, *Gli1*, *ptch2* for SHH pathway; *Cdk4*, *Ccne1*, *Aurkb* for cell cycling) that are known to regulate follicle stem cells showed extensive H3K27ac modifications (Supplemental Figure 1A). This finding suggests that, in HFSCs, activation of those genes may critically depend on BRD4, which acts as a key chromatin reader recognizing the acetylated H3K27 to activate gene transcription (38–40). Thus, in the absence of BRD4, the follicle stem cells may fail to activate those signaling pathways and, consequently, fail to proliferate and die. These events collectively result in the collapse of hair follicle and the emergence of alopecia.

Another important finding is that the tissue pathology in *Brd4^fl/fl^ Ox40-Cre* mice is restricted to the skin, as other tissues and organs did not show any signs of local inflammation. The skin inflammation involves the activation of γδ T cells, and their activation and the subsequent production of inflammatory cytokines depend on the death of follicle stem cells, thus highlighting a complex network of cellular interactions in the induction of dermatitis. In a series of experiments involving adoptive T cell transfer, chimeric mice, and the *Rag1^–/–^* mice where *Brd4* was deleted in HFSCs, we demonstrated that deletion of *Brd4* in the *Rag1^–/–^* hosts abrogated hair growth and regeneration without the induction of dermatitis, but adoptive transfer of γδ T cells into those mice readily capitulated dermatitis as observed in *Brd4^fl/fl^ Ox40-Cre* mice. However, the same γδ T cells transferred into unmanipulated *Rag1^–/–^* mice, which have normal healthy hair stem cells, failed to trigger dermatitis (Figure 6). It is well known that the skin hosts large numbers of γδ T cells that are highly responsive to changes in their local milieu (i.e., sentinel cells) (41). Thus, death of follicle stem cells may activate local γδ T cells through the release of damage associated molecules (42, 43), which further drives skin inflammation. Interestingly, the γδ T cells are also the targets of Tregs, where functionally capable Tregs could inhibit the activation of γδ T cells, as well as γδ T cell–mediated skin inflammation (Figure 7). As Tregs constitutively express OX40, and *Ox40-Cre*–mediated deletion of *Brd4* in Tregs compromised their suppressive functions, this Treg defect in *Brd4^fl/fl^ Ox40-Cre* mice allowed hyperactivation of γδ T cells in situ, thus further contributing to the development of dermatitis and hair follicle destruction.

The exact mechanisms of how BRD4 regulates Tregs remain to be defined in future studies. But recent studies suggest a fundamental role of chromatin modifications, even before the expression of Foxp3, in the development of Foxp3^+^ Tregs (44). In fact, Sakaguchi and colleagues recently reported that the pioneering factor Satb1 critical to Treg induction is under the control of super enhancers (45). Thus, it is conceivable that the defects in Tregs following *Brd4* deletion may be due to the disruption of super enhancer elements. Similarly, the cell types in the skin hair follicles that express the ligand for OX40 (OX40L) that engages OX40 on follicle stem cells are also unknown. In the immune system, OX40L is abundantly expressed by antigen-presenting cells, especially DCs (46); whether similar cell types are present in the hair follicles, particularly during skin inflammation, remains to be defined. Additionally, a better understanding of the enhancer landscapes in follicle stem cells, especially in different phases of activation, and the precise roles of chromatin modifiers involved will have major implications in management of skin diseases, including alopecia.

Our studies may have important clinical implications in several disease settings. Preserving OX40 signaling and BRD4 functions is critical in hair follicle stem cell fitness, survival, and proliferation, which has implications in treatment of hair loss and alopecia. Thus, therapies that either block OX40 (in autoimmune diseases, transplant rejection) or stimulate OX40 signaling (e.g., cancer immunotherapies) may have unintended complications in the skin. Also, γδ T cells are key mediators in inflammatory skin diseases, including psoriasis, as they are major sources of the inflammatory cytokine IL-17 (47, 48). γδ T cells are highly sensitive to changes in the local environment, including death of follicle stem cells; targeting the activation of γδ T cells and/or their inflammatory products is a viable therapeutic approach in treatment of skin inflammation. Furthermore, considering the role of BRD4 in Tregs, modulating Treg functions by targeting BRD4 may have important therapeutic implications in autoimmune diseases and cancer therapies.

## Methods

### Mice.

*Brd4^fl/fl^*, *Ox40-Cre*, *Cd4-Cre*, *Rosa26^fl/fl^-Yfp*, *Rag1^–/–^*, and *CD45.1* congenic mice were obtained from The Jackson Laboratory. *Ox40-Cre* and *Cd4-Cre* mice were bred with *Brd4^fl/fl^* mice to generate *Brd4^fl/fl^ Ox40-Cre* and *Brd4^fl/fl^Cd4-Cre* mice at our animal facility. *Rosa26^fl/fl^-Yfp* mice were crossed with *Ox40-Cre* mice to generate *Rosa26^fl/fl^-Yfp–Ox40-Cre* mice. *Rag1^–/–^* mice were crossed with *Ox40-Cre* mice to generate *Rag1^–/–^ Ox40-Cre* mice, which were further bred with *Brd4^fl/fl^* mice to generate *Rag1^–/–^* CKO mice. *CD45.1* congenic *Foxp3gfp* mice were bred to harvest GFP-tagged Tregs by cell sorting for the purpose of adoptive cell transferring. All animals were maintained in specific pathogen–free conditions at Houston Methodist Hospital.

### Disease scoring.

The skin diseases were scored based on 2 aspects: hair loss and dermatitis conditions. The degree of hair loss was recorded as a relative percentage over healthy control mice, where no hair loss was designated as 0; 1, < 30% hair loss; 2, 30%–70% hair loss; and 3, > 70% hair loss. For dermatitis scoring, each of the following signs was assessed: skin dryness, thickening, crusting, and eruption as 0, none; 1, mild; 2, moderate; and 3, severe. The scores were added and presented in line graphs over time. The total disease score was the sum of 2 individual attributes with the maximum combined score as 6 in each group.

### Hair growth cycle analysis.

Mouse hair growth cycles were documented by photographs after birth. Anagen was determined by darkening of the skin followed by hair growth. The back skin of mice was shaved with an electric clipper to reveal skin color changes and hair coat recovery. Once the hair coat recovery reached about 90% of the back skin, the mice were shaved again to monitor the entry into the next anagen phase. To assess the length of each hair cycle phase (telogen, anagen, catagen), skin color changes were documented every 2–3 days.

### Isolation and analysis of HFSCs.

For isolation of HFSCs, mouse dorsal skin was dissected, and the fat layer was removed using a surgical scalpel. The skin was incubated in trypsin (Thermo Fisher Scientific, 15090-046; 0.25%) for 1.5 hours in a 37°C incubator. A single-cell suspension was obtained by scraping the upper side and filtering. Cells were centrifuged for 5 minutes at 500*g* at 4°C, resuspended in DMEM with 5% FBS, and stained for 15–20 minutes. The following antibodies were used for surface staining: CD49f (Itgα6), CD34, Sca-1, and CD45 (Table 1). The HFSCs were identified as CD45^−^Itgα6^+^CD34^+^Sca-1^−^ cells.

### Tissue histology and immunofluorescence.

All H&E staining and TUNEL staining were performed by the Human Tissue Acquisition and Pathology core at Baylor College of Medicine (Houston, Texas, USA). For immunofluorescence, fresh skin samples were embedded in OCT compound (Thermo Fisher Scientific, 4585) overnight at –80°C. The next day, the embedded samples were processed as frozen sections to approximately 10 μm and then fixed in 4% paraformaldehyde (PFA; Invitrogen, FB002) for 15 minutes at room temperature and immersed twice in PBS for 5 minutes each time. The slides were then blocked with blocking buffer (5% FBS and 0.2% Triton X-100 in PBS) for 1 hour and incubated with primary antibodies overnight in humid box at 4°C. The next day, the slides were immersed in blocking buffer for 5 minutes and incubated with secondary antibodies for 2 hours at room temperature. The slides were washed twice in PBS for 5 minutes each time and mounted with mounting agent (Fluoromount-G, with DAPI; Invitrogen, 00-4959-52). The following antibodies and dilutions were used: keratin 14 (Santa Cruz Biotechnology Inc., sc53253, 1:200), CD3 (BioLegend, 100236, 1:200), IL-17A (BioLegend, 506904, 1:200), Alexa Fluor Plus 488 (Invitrogen, A32723, 1:300), and Alexa Fluor Plus 647 (Invitrogen, A32728, 1:300). DAPI was used as a counterstain for the nucleus.

### Flow cytometry and cell sorting.

For peripheral circulating lymphocytes, single-cell suspensions were prepared from spleens or lymph nodes by mechanical disruption and filtration. RBCs were lysed with ACK Lysing Buffer (Thermo Fisher Scientific, A10492-01). For skin-specific lymphocytes, mouse dorsal skin was harvested and minced with scissors, and it was then digested with Collagenase I (MilliporeSigma, SCR103; 3 mg/mL in DMEM) and DNase I (Invitrogen; 60μg/mL) at 37°C for 1 hour on an orbital shaker. Single-cell suspensions were obtained by filtration and gradient centrifugation using 44% Percoll buffer (Percoll PLUS, GE Healthcare, 17-5445-01).

For surface staining, single-cell suspensions were first stained with Zombie Aqua Viability Dye (BioLegend), followed by Fc blocking with anti–mouse CD16/32 (eBioscience, 14-0161-82), and then stained with the appropriate antibodies. For intracellular cytokine staining, CD4 and γδ T cells were stimulated for 4 hours with phorbol 12-myristate 13-acetate (50 ng/mL) and ionomycin (550 ng/mL; Sigma-Aldrich) in the presence of GolgiStop (BD Pharmingen). Cells were then fixed and permeablized with Foxp3 staining buffer set (Invitrogen, 00-5523-00) or BD Phosflow reagents (catalogs 557870 and 558050) to preserve the YFP sign, and they were then stained with antibodies as indicated according to manufacturer instructions. For flow cytometry analysis, all data were acquired using LSRII. Cell sorting was performed on BD FACSAria instrument. Data were analyzed with FlowJo v10 software. Antibodies used in Flow cytometry and cell sorting are listed in Table 1.

### BrdU treatment.

The back skin of *Rag1^–/–^* and *Rag1^–/–^* CKO mice was shaved on P20 to ensure hair cycle at the telogen stage. BrdU (BioLegend; 50 mg/kg) was administered by i.p. injection. BrdU was given at days 20, 21, and 22 consecutively before the hair cycle entered anagen stage to allow a chase period until hosts’ back skin darkened. BrdU-labeled cells were analyzed on P28.

### Adoptive cell transfer and BM chimera.

Total T cells were purified from the spleens and lymph nodes of *Ox40-Cre* or *Brd4^fl/fl^ Ox40-Cre* mice by mouse depletion Dynabeads (Invitrogen, 11413D). γδ T cells were further sorted using FACS. Total T cells (10 × 10^6^ per mouse) or γδ T cells (1 × 10^6^ per mouse) were transferred to *Rag1^–/–^* or *Rag1^–/–^* CKO recipients via tail vein injection. For BM chimeric experiments, BM cells isolated from the femurs and tibia of *Ox40-Cre* or *Brd4^fl/fl^ Ox40-Cre* mice were transferred i.v. into *Rag1^–/–^* or *Rag1^–/–^* CKO recipients irradiated with 5 Gy using an RS2000 irradiator (Red Source Technologies Inc.) 4 hours before BM cell transfer.

### qPCR and RNA-Seq.

HFSCs from *Rag1^–/–^* or *Rag1^–/–^* CKO mice were sorted, spun down, and dissolved in TRIzol (Invitrogen, 10296028), and total RNA was harvested by means of the Direct-zol RNA MicroPrep Kit (Genesee Scientific, 11-330MB). Total RNA quality was determined with a spectrophotometer (Thermo Fisher Scientific, NanoDrop 2000). cDNA was then synthesized using iScript Reverse Transcription Supermix (Bio-Rad, 1708841), and qPCR was performed using SYBR Green Supermix (Bio-Rad, 1725275). Data were normalized to the expression of housekeeping GAPDH genes, and the relative abundance of transcripts was calculated by the 2^–ΔCT^ method. All primer sequences are listed in Table 2.

For RNA-Seq studies, HFSCs were sorted on day 20, day 26, and week 10 after the mice were born. For all samples, polyA^+^ RNA was isolated and RNA-Seq analysis was performed by BGI. Heatmaps, KEGG pathways, and GSEA were generated using Dr. Tom platform (https://biosys.bgi.com/). The RNA-Seq data sets were deposited in the NCBI’s Gene Expression Omnibus (GEO) database (GSE213603).

### Statistics.

Statistical analysis was performed using GraphPad Prism software, version 7. For 2-group comparisons, 2-tailed Student’s *t* test was used to generate *P* values. Multiple sample comparisons were made using 1-way ANOVA. For all statistical tests, *P* < 0.05 was defined as statistically significant.

### Study approval.

All animal studies were reviewed and approved by the IACUC at Houston Methodist Hospital, Texas Medical Center, in accordance with institutional guidelines.

## Author contributions

MW, YY, and XX designed the studies, performed experiments, and tabulated the data. PRA, GW, and XC assisted in experiments and data interpretation. RMG provided helpful discussions. XCL, MW, and XX wrote the manuscript. XCL supervised the project and finalized the manuscript.

## Supplementary Material

Supplemental data

## Figures and Tables

**Table 1:**
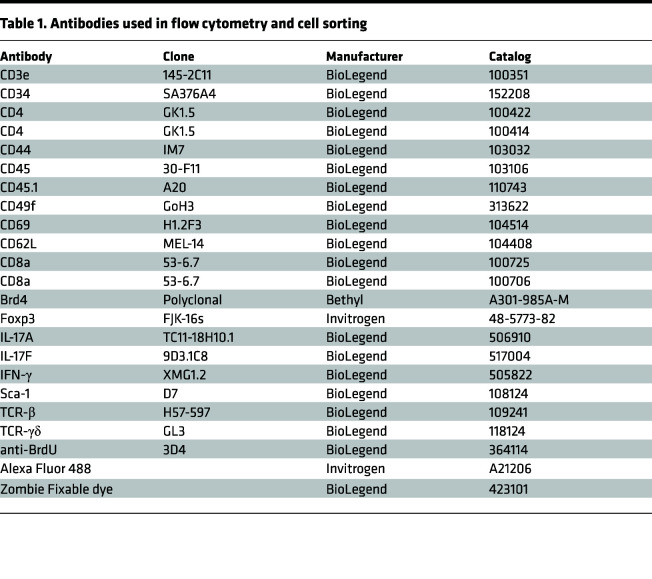
Antibodies used in flow cytometry and cell sorting

**Table 2:**
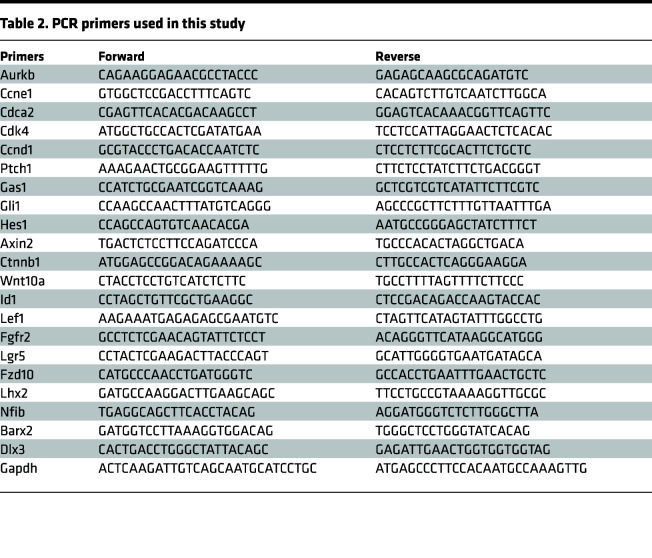
PCR primers used in this study

## Version 1 10/18/2022

In-Press Preview

## Version 2 12/08/2022

Electronic publication
