# Supplementary material for: Ox40-Cre–mediated deletion of BRD4 reveals an unexpected phenotype of hair follicle stem cells in alopecia
Source: JCI Insight. 2022 Dec 8;7(23):e164534. doi: 10.1172/jci.insight.164534 (PMC9746908; doi:10.1172/jci.insight.164534)
Supplement: Supplemental data [file jciinsight-7-164534-s062.pdf]

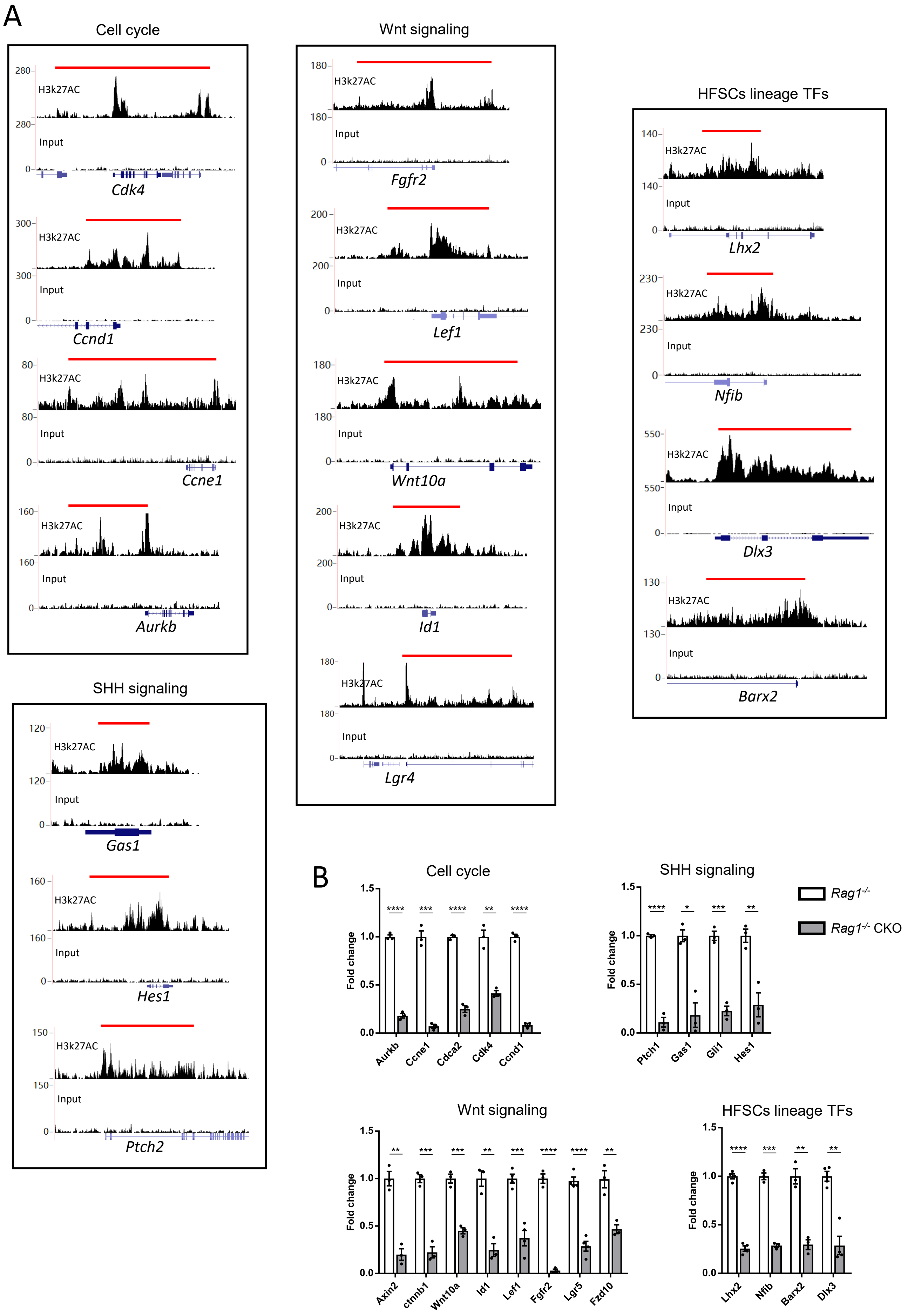

Supplemental Figure 2

Supplemental figure 1, related to figure 5. (A) H3k27ac ChIP-seq peaks in wildtype mice in genes related to cell cycle, SHH signaling, Wnt signaling and follicle stem cell lineage transcription factors using telogen HFSCs. These data are quoted from GSM1501996, GSM1501997. **(B)** qRT-PCR of genes related to cell cycle, SHH signaling, Wnt signaling and HFSCs lineage transcription factors using telogen follicle stem cells from $Rag1^{-/-}$ and $Rag1^{-/-}$ CKO mice. $n = 5$-8. The data are representative of at least three independent experiments and are shown as mean $\pm$ SD. $^{*}P < 0.05$, $^{**}P < 0.01$, $^{***}P < 0.001$, $^{****}P < 0.0001$, by two-tailed Student's *t* test.

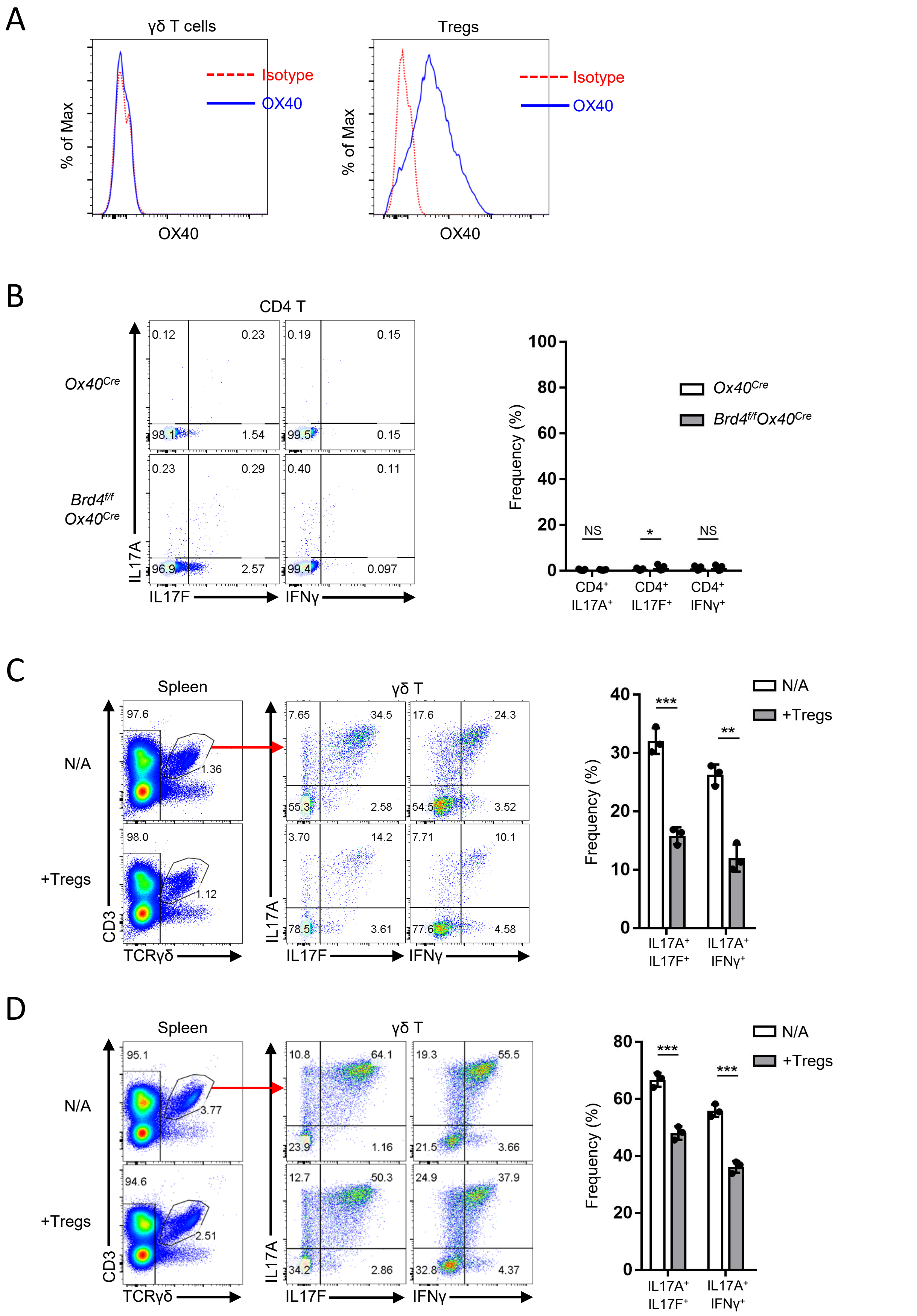

Supplemental Figure 2

Supplemental figure 2, related to figure 6 and 7. (A) Graph shows OX40 expression on γδ T cells (left) compared with Tregs (right, as positive control). **(B)** Flow cytometry analysis of intracellular expression of cytokines (IL17A, IL17F and IFNγ) in $CD4^+$ cells from the skin of control *Ox40-Cre* and *Brd4*$^{f/f}$*Ox40-Cre* mice on postnatal day 84 (left), and frequency of cytokines are indicated (right). *n* = 5. **(C)** Flow cytometry analysis of intracellular expression of cytokines (IL17A, IL17F and IFNγ) in γδ T cells from spleen of $CD45.1^+$ Tregs recipients compared with N/A mice at 14 weeks old (left), and frequency of IL17A+IL17F+ or IL17A+IFNγ+ cells (right). *n = 3*. **(D)** Flow cytometry analysis of intracellular expression of cytokines (IL17A, IL17F and IFNγ) in γδ T cells from peripheral lymph nodes of $CD45.1^+$ Treg recipients compared with N/A mice at 14 weeks old (left), and frequency of IL17A+IL17F+ or IL17A+IFNγ+ cells (right). *n = 3*. Data are shown as mean ± SD. *$P < 0.05$, **$P < 0.01$, ***$P < 0.001$, *NS*, not significant, by two-tailed Student's *t* test.
